# Intranasal Immunization with an Archaeal Lipid Mucosal Vaccine Adjuvant and Delivery Formulation Protects against a Respiratory Pathogen Challenge

**DOI:** 10.1371/journal.pone.0015574

**Published:** 2010-12-29

**Authors:** Girishchandra B. Patel, Hongyan Zhou, Amalia Ponce, Greg Harris, Wangxue Chen

**Affiliations:** Institute for Biological Sciences, National Research Council Canada, Ottawa, Canada; Statens Serum Institute, Denmark

## Abstract

Archaeal lipid mucosal vaccine adjuvant and delivery (AMVAD) is a safe mucosal adjuvant that elicits long lasting and memory boostable mucosal and systemic immune responses to model antigens such as ovalbumin. In this study, we evaluated the potential of the AMVAD system for eliciting protective immunity against mucosal bacterial infections, using a mouse model of intranasal *Francisella tularensis* LVS (LVS) challenge. Intranasal immunization of mice with cell free extract of LVS (LVSCE) adjuvanted with the AMVAD system (LVSCE/AMVAD) induced *F. tularensis*-specific antibody responses in sera and bronchoalveolar lavage fluids, as well as antigen-specific splenocyte proliferation and IL-17 production. More importantly, the AMVAD vaccine partially protected the mice against a lethal intranasal challenge with LVS. Compared to LVSCE immunized and naïve mice, the LVSCE/AMVAD immunized mice showed substantial to significant reduction in pathogen burdens in the lungs and spleens, reduced serum and pulmonary levels of proinflammatory cytokines/chemokines, and longer mean time to death as well as significantly higher survival rates (*p*<0.05). These results suggest that the AMVAD system is a promising mucosal adjuvant and vaccine delivery technology, and should be explored further for its applications in combating mucosal infectious diseases.

## Introduction

Many microbial pathogens invade their human and animal hosts through the mucosal surfaces of the respiratory, gastrointestinal and urogenital tracts [1], [2]. Immunity at the mucosal surface would help prevent the pathogen from establishing and from disseminating to other organs to cause systemic disease. The majority of currently approved human/veterinary vaccines are administered systemically, and they fail to elicit effective mucosal immunity [2], [3], [4]. The few mucosal vaccines currently in the marketplace are all based on the use of live-attenuated or dead pathogen cells [3], [5], [6], [7]. Although these vaccines are efficacious, there are lingering concerns regarding potential reversion to virulence, overall safety in immunocompromised populations [3], [8], [9], and the possible inclusion of toxic cell components such as endotoxins [10]. Vaccines based on acellular or subunit antigens would be safer, but such antigens are generally poorly immunogenic on their own [3], [10], [11]. This has sustained global research efforts at developing mucosal adjuvants and non-replicating delivery systems such as detoxified cholera toxin (CT) and *Escherichia coli* heat labile toxin, CpG oligonucleotides, DNA, microparticulates such as virosomes, liposomes, cochleates, polymeric microspheres, and immunostimulating complexes such as ISCOMs [8], [9], [11], [12].

We recently demonstrated that intranasal (i.n.) immunization of mice with ovalbumin (OVA) formulated in archaeal lipid mucosal vaccine adjuvant and delivery (AMVAD) structures prepared from the total polar lipids extract (TPL) of *Methanobrevibacter smithii* (OVA/AMVAD), or other archaeal species, elicited strong anti-OVA IgA responses at both local (nasal) and distal (gastrointestinal and vaginal) sites, and in sera [13]. Additionally, robust, antigen-specific systemic antibody (serum IgG1 and IgG2a) and CD8^+^ CTL responses were also generated. The mucosal and systemic responses elicited were generally well sustained over time, and exhibited strong memory boost responses. Detailed toxicity evaluation in mice demonstrated an excellent safety profile for the AMVAD system at an i.n. dose that was 10-fold greater than that required for vaccine efficacy [14]. These results suggested that the AMVAD system represents a promising technology for mucosal vaccine development. However, the potential of the AMVAD system in eliciting protection against an infectious challenge had not been evaluated to-date.

In the current study, using a mouse model of i.n. challenge with *Francisella tularensis* live vaccine strain (LVS), we show that the AMVAD based vaccine induced antigen-specific cellular and humoral immune responses, reduced the tissue pathogen burdens, and enhanced the survival of the challenged mice, compared to the naïve mice or the mice immunized with the antigen alone.

## Methods

### Total polar lipids extrac*t*

The archaeal species *Methanobrevibacter smithii* ALI (DSM 2375) was grown in a 75 L fermenter vessel as described previously [15]. The total polar lipids extract (TPL) was obtained from the biomass by solvent extraction [16]. The TPL was analyzed by FAB MS and thin layer chromatography for quality control purposes and was stored in chloroform, at 4°C to minimize solvent evaporation.

### *Francisella tularensis LVS* cell free extract (LVSCE) antigen preparatio*n*

*Francisella tularensis* LVS (ATCC 29684) cells grown on 40 plates of cysteine heart agar supplemented with 1% wt/vol haemoglobin and 1% vol/vol of Isovitalex^R^ enrichment (Beckton and Dickinson, Sparks, MD, USA) were harvested, washed, and re-suspended into 160 ml of saline (0.85% NaCl, autoclaved 121°C for 15 min). The cells were lysed by two successive passages (68,900–103,350 KPa) through an Emulsiflex^R^-C5 high pressure homogenizer (Avestin Inc., Ottawa, Ontario, Canada). The lysate was centrifuged at 16,000×*g* for 90 min, the supernatant containing the cell-free extract (LVSCE) was filtered through 0.22 µm filters, and an aliquot was plated on cysteine heart agar to verify absence of viable cells. The total protein content of the LVSCE was 4.46 mg/ml by Lowry assay, using bovine serum albumin as the standard, and was stored at 4°C till used.

### Preparation and characterization of LVSCE/AMVAD formulation

The LVSCE/AMVAD formulation was prepared aseptically, using pyrogen-free glassware and sterile Milli-Q^R^ water. Empty, small (ca 100 nm average diameter), unilamellar archaeosomes (i.e., liposomes made from archaeal polar lipids) were prepared by hydration of 20 mg TPL in 1.0 ml water (at room temperature), as described previously [17]. The archaeosome suspension was supplemented with 22.4 µl of LVSCE (0.1 mg total protein), and the total volume was made up to 1.9 ml by adding saline. While vigorously vortexing the LVSCE/archaeosome suspension in the presence of 5 sterile glass beads (ca 3 mm diameter each) to aid mixing, 0.1 ml of 1.0 M filter sterilized stock CaCl_2_ solution was added in a drop-wise manner to convert the suspension into LVSCE/AMVAD formulation, as described previously for making OVA/AMVAD formulations [14], [17]. The LVSCE/AMVAD formulation was further vortexed for approximately 3 min to reduce the average width of >95% of the AMVAD structures to less than 5 µm. The LVSCE/AMVAD preparation was viewed under phase contrast microscopy (ca 1250× magnification) to verify that the typical, individual, very small, spherical archaeosome structures (barely visible at this magnification) in the original LVSCE/archaeosome suspension were absent or very minimal, and had been predominantly converted into much larger aggregates with phase bright surface perimeters [13], [17] which represent typical AMVAD structures. The appearance of AMVAD formulation under phase contrast microscopy was recorded using an Olympus Model BX51 TF microscope (Olympus America, Melville, NY, USA) mounted with a Micropublisher^R^ 5.0 RTV digital camera (QImaging, Burnaby, British Columbia, Canada). The average width of the AMVAD structures in the formulation was determined by randomly measuring the widths of a minimum of 100 AMVAD structures from the images taken above, using QCapture Pro software (QImaging).

Based on the starting amount of the lipid used for making the archaeosomes, the total LVSCE protein added, and amount of the CaCl_2_ added to make the LVSCE/AMVAD formulation, the ratio of antigen∶lipid (w/w) was 1∶200, the ratio of lipid∶Ca^2+^ (w/w) was 1∶5, and the CaCl_2_ concentration in the formulation was 50 mM.

All LVSCE/AMVAD formulations were stored at 4°C until use. Just prior to use for each immunization, aliquots of the AMVAD formulation were diluted to the immunization dose in a final concentration of 0.85% saline/20 mM CaCl_2_ (pH 7.1).

### Mice immunizations and pre-challenge sample collection

The efficacy of LVSCE/AMVAD vaccine in eliciting mucosal and systemic immune responses, and in affording protection against an i.n. pathogen challenge was evaluated in BALB/c mice. Specific-pathogen-free, female BALB/c mice were purchased from Charles Rivers Laboratories (Montreal, QC, Canada), and entered the experiments at 6–8 weeks of age (about 18 g). Mice were housed and used as per the Canadian Council on Animal Care Guide to the Care and Use of Experimental Animals. This study and all animal care/use protocols were approved (ID # 2007-15) by the Institute for Biological Sciences (National Research Council Canada) Animal Care Committee.

Groups of mice (n = 20) were immunized intranasally (50 µl volume) after anesthetizing with isofluorane. The mice were immunized (0, 7 and 21 d) with LVSCE alone (in saline), LVSCE admixed with 1.0 µg cholera toxin (LVSCE/CT, Sigma-Aldrich Canada Ltd, Oakville, Ontario, Canada), or LVSCE/AMVAD formulation. A fourth group consisted of naïve mice. The LVSCE antigen dose (total protein basis) in each instance was 1 µg/mouse/immunization. For the LVSCE/AMVAD vaccine, this antigen dose corresponded to the inclusion of 0.2 mg of the archaeal TPL as part of the LVSCE/AMVAD formulation.

At day 28, 5 mice per group were euthanized by CO_2_ asphyxiation and the sera were obtained (for anti-body assays and cytokines) from total blood collected by cardiac puncture [13], and the spleens were harvested (for *in vitro* splenocyte proliferation assay). The lungs were lavaged 5 times with 1.0 ml PBS containing 3 mM EDTA [18] to obtain bronchoalveolar lavage (BAL) fluid. A haemocytometer was used to count the total BAL cells. Cytospin slides were prepared and stained with HemaStat 3^R^ (Fisher, Pittsburgh, PA), and 200 cells were examined to determine the differential cell counts. The rest of the BAL fluid was centrifuged (2,450×*g*, 7 min) and the supernatant was stored at −80°C until analyses.

### ELISA for LVSCE-specific IgA, IgM, IgG and IgG isotypes

LVSCE-specific IgA, IgM, IgG1, and IgG2a antibodies were measured by indirect enzyme-linked immunosorbent assays (ELISA) [13]. Briefly, 96-well flat-bottom Immunolon 2^R^ microplates (Thermo Electron Corporation, Milford, MA, USA) were pre-coated with 0.5 µg LVSCE/well, in 100 µl of 0.1 M bicarbonate buffer (pH 9.6). Serially diluted (2-fold increments) samples of sera and BAL fluid, as indicated in the figures, were used to derive the antibody titration curves.

### Determination of antigen-specific splenocyte proliferation and cytokine production

In selected experiments, the immunized and naïve mice were sacrificed at day 28 and their spleens aseptically removed and used to prepare single cell suspensions. Spleen cells were suspended at a concentration of 2.5×10^6^ cells/ml in Dulbecco's Modified Eagle Medium (DMEM) containing 2 mM L-glutamine, 25 mM HEPES, 10% fetal bovine serum, 5×10^−5^ M 2-mercaptoethanol, 100 U of penicillin/ml, and 100 µg of streptomycin/ml, in the presence of formalin-fixed *F. tularensis* LVS (ffLVS; 2×10^6^ bacterial cells/ml), or Concanavalin A (Con A, 5 µg/ml) as the positive control for the assay, or medium only (as the negative control for the assay), as described previously [18]. The cells were cultured (37°C, 5% CO_2_) in duplicates in 24-well (for culture supernatant), or in triplicates in 96-well (for proliferation assay), flat-bottom tissue culture plates. For spleen cell proliferation assay, the cells were cultured for 90 h. At 72 h, 1 µCi of ^3^H-thymidine was added to each well, and the cells were harvested at the end of the culture period and analyzed for ^3^H-thymidine incorporation using a beta-scintillation counter. Stimulation Index was calculated as [counts per minute, stimulated cells]/[counts per minute, media-treated control cells]. For cytokine measurements, cell culture supernatants were collected at 48 h, centrifuged, and stored at −80°C until assay.

### Cytokines/chemokines measurement

The cytokine/chemokine levels in sera, BAL fluid, and the supernatants of lung homogenates and spleen cell cultures were measured in duplicate, using Milliplex MAP^R^ mouse cytokine/chemokine kit (Millipore, Billerica, MA, USA) and a Luminex® 100IS system (Luminex Corp., Austin, TX). The cytokine/chemokine concentrations were calculated against the standards, using Beadview^R^ software (Upstate Group LLC, Lake Placid, NY) [19].

### Intranasal challenge with *F. tularensis* LVS and monitoring

At day 35 (14 day post the last immunization) mice were anesthetized by i.p. injection of ketamine and xylazine (at 0.1 mg and 0.05 mg/g body weight of mouse, respectively, in 0.25 ml injectable saline) and intranasally challenged with 3.4×10^4^ colony forming units (CFU) of *F. tularensis* LVS in 50 µl saline. In our laboratories, the LD_50_ upon i.n. administration of this strain to female BALB/c mice is ca 10^3^ CFU. Each group of mice was then split into three sub-groups of 5 mice each. In addition to the availability of the normal sources of food and water ad labium, all the challenged mice were given access to “Nutra-gel” (Bio-Serv, Frenchtown, NJ, USA) tablets which were placed on the floors of the cages.

The body weights of one sub-group of each of the 4 challenged groups of mice were monitored once daily and the clinical signs were monitored and recorded. The body weight change was calculated as a percentage change from the pre-challenge weight. The overall clinical sign for each mouse was scored on a sliding scale of 0 to 5. Individual clinical scores were assigned as 0 (normal, active, healthy), 1 (slightly sick, slightly ruffled fur, otherwise normal), 2.0 (sick, ruffled fur, slow movement, hunching), 3.0 (very sick, ruffled fur, very slow movement, hunched, eyes shut), 4.0 (moribund), or 5 (dead). To calculate the average clinical score of a sub-group of mice, any mouse that had died (or had been euthanized for humane reasons) on that day was included in the averaging of the clinical score for that day, but not for averaging the score on subsequent days for the surviving mice in the group. The % of mice in each group that survived the challenge was recorded.

### Pathogen burdens and post-challenge sample analyses

At 1 and 4 days post i.n. challenge, 5 mice per each group were euthanized and the sera were collected and assayed for cytokines/chemokines as described above. The lungs and spleens were homogenized in 2 ml saline and 10-fold serial dilutions were plated on chocolate agar plates supplemented with haemoglobin, Isovitalex^R^ and antibiotics [18], to determine the *F. tularensis* LVS burdens in the respective organs. Remainder of the lung homogenate was treated with Complete® protease inhibitor (Roche Diagnostics, Laval, QC, Canada) and the supernatant collected (2,450×*g*, 7 min) for cytokine/chemokine assays as described above.

### Statistical analyses

Data are presented as means ± SD. Differences between groups were determined by one- or two-way analysis of variance (ANOVA) followed by Dunnett's multiple comparison test or Bonferroni post test, respectively (GraphPad Prism 4.0, GraphPad Software, San Diego, CA). The survival curves data were statistically analyzed by the logrank test. Differences were considered significant at *p*<0.05.

## Results

### Vaccine induced mucosal and systemic antibody responses

**Figure 1:**
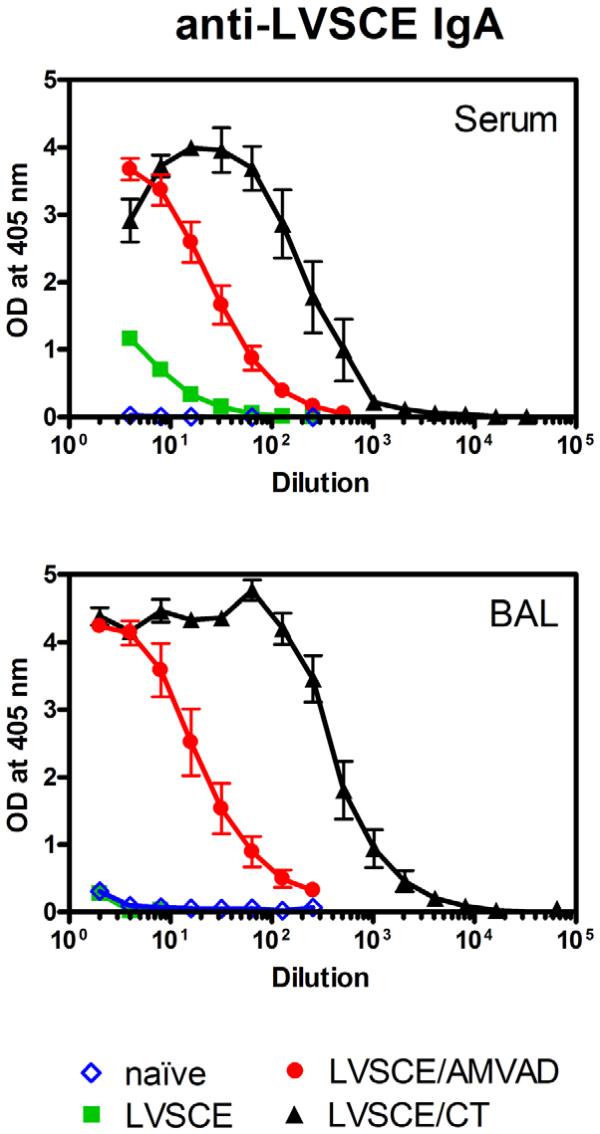
LVSCE/AMVAD vaccine induces strong LVSCE-specific IgA levels in sera and BAL fluids. Groups of female BALB/c mice (n = 20) were immunized i.n. at 0, 7 and 21 d (1 µg LVSCE antigen, on total protein basis, per mouse per immunization) with LVSCE, LVSCE/AMVAD or LVSCE/CT. A naïve control group was also included. Five mice per group were euthanized at 28 d, for collection of sera and BAL fluids for LVSCE-specific IgA analyses by ELISA. Each data point represents the mean OD ± SD for each group. Data are representative of two separate experiments.

**Figure 2:**
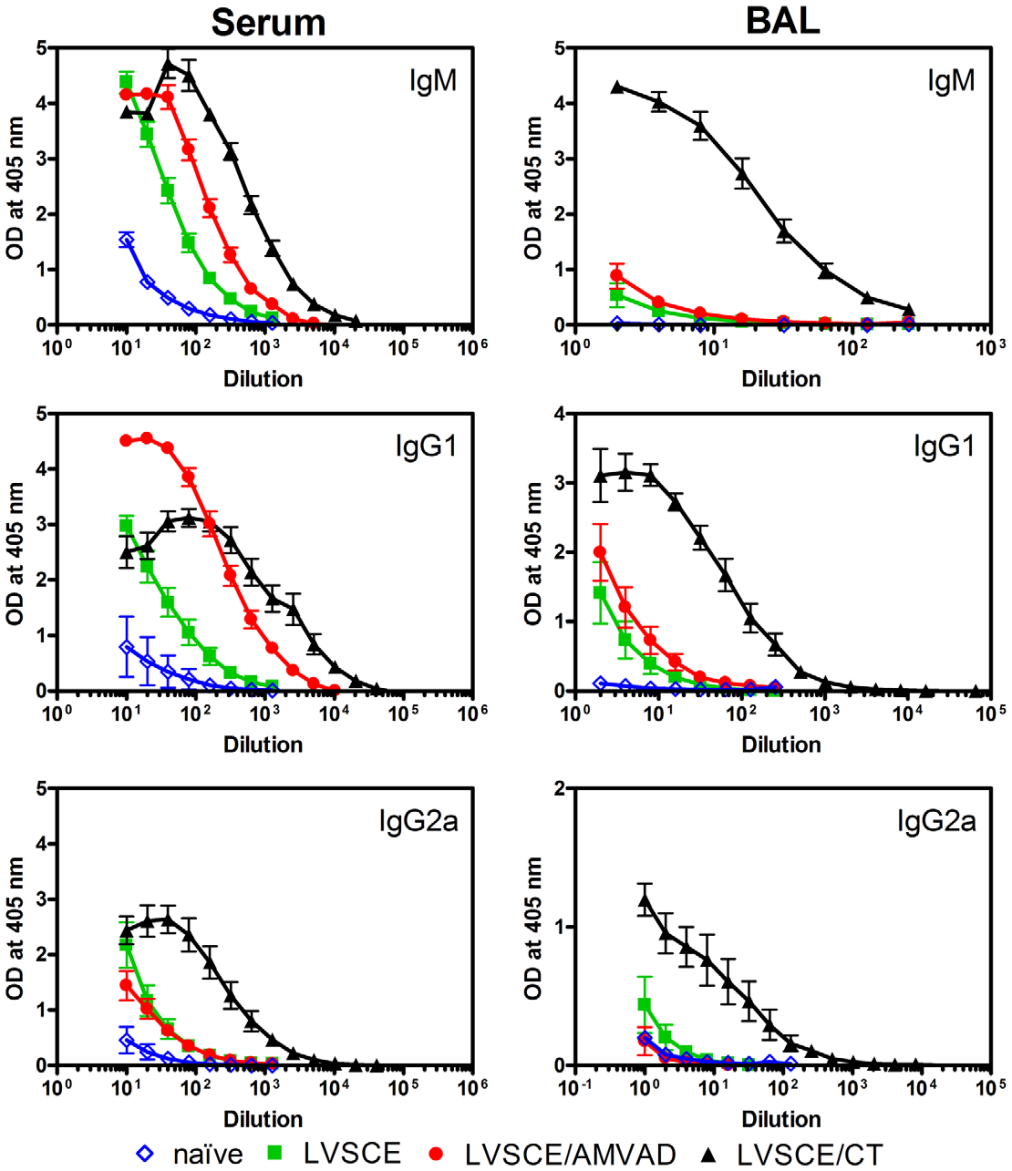
LVSCE/AMVAD vaccine induces strong LVSCE-specific IgM, IgG1 and IgG2a levels in sera and BAL fluids. The sera and BAL fluids collected at 28 d from the naïve, and LVSCE, LVSCE/AMVAD or LVSCE/CT immunized groups of mice in Figure 1 were analyzed for the indicated LVSCE-specific antibody by ELISA. Each data point represents the mean OD ± SD for each group (n = 5). Data are representative of two separate experiments.

The AMVAD system is a non-replicating mucosal adjuvant/delivery system. Therefore, we used a vaccine adjuvanted with CT (LVSCE/CT) as a positive control in our study, since it is recognized as a strong, non-replicating mucosal adjuvant. However, the toxicity of CT in humans precludes its use in vaccines for humans. The other controls included antigen alone (LVSCE) immunization, and naïve mice. The anti-LVSCE IgA antibody titres measured in the sera and BAL fluids of mice immunized with the LVSCE/AMVAD vaccine were strong and much higher than those seen with LVSCE alone immunization or in naïve mice (Fig. 1). As expected, the strongest responses were in mice immunized with LVSCE/CT. The systemic immune responses assessed as anti-LVSCE IgM and IgG1 antibody titres in sera from mice immunized with LVSCE/AMVAD and LVSCE/CT vaccines were strong, and generally comparable (Fig. 2). However, the specific IgM and IgG1 responses in the BAL fluids, and the specific IgG2a titres in sera and BAL fluids, were the strongest in the LVSCE/CT group.

### Vaccine induced antigen-specific cellular immune responses

**Figure 3:**
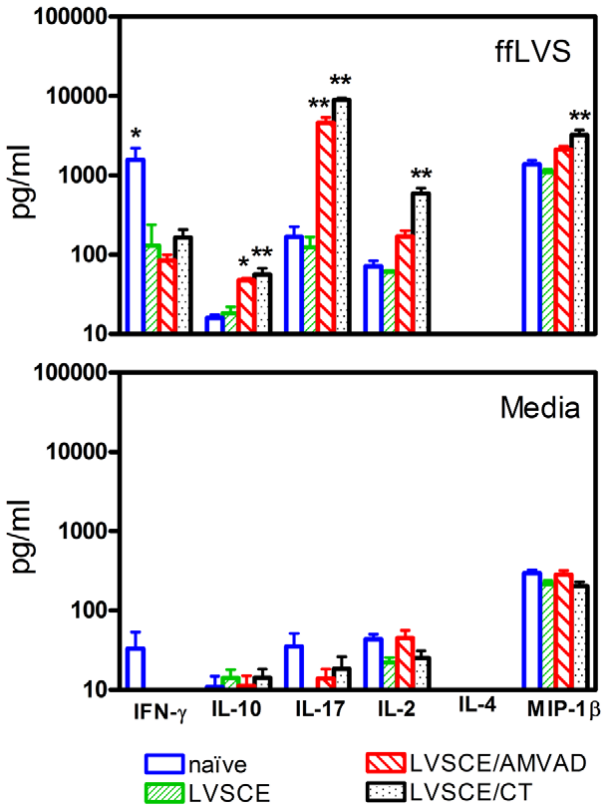
LVSCE/AMVAD vaccine induces production of cytokines by splenocytes. Splenocytes (2.5×10^6^ cells/ml) obtained from the naïve, and LVSCE, LVSCE/AMVAD or LVSCE/CT immunized mice euthanized at 28 d in Figure 1 experiment, were stimulated *in vitro* for 48 h (37°C, 5% CO_2_) in the presence of formalin-fixed *F. tularensis* LVS (ffLVS; 2×10^6^ cells/ml). The supernates were collected and assayed for various cytokines. Supernates collected from the respective splenocytes stimulated with media only were the negative controls, and those collected from splenocytes stimulated with Concanavalin A (data not shown) were the positive controls, respectively, for the assay. The data are presented as mean ± SD for each group (n = 5) of naïve, and LVSCE, LVSCE/AMVAD or LVSCE/CT immunized mice. ^*^ *p*<0.05 and ^**^ *p*<0.01 vs. LVSCE alone immunized group.

To determine the antigen-specific cellular immune responses elicited by LVSCE/AMVAD vaccine, we assessed the antigen-specific lymphocyte proliferation response and the production of IFN-γ, IL-2, IL-4, IL-10, IL-17 and MIP-1β by the splenocytes, in response to stimulation with formalin-fixed *F. tularensis* LVS. The splenocytes from LVSCE/AMVAD-immunized mice showed higher (but not significantly) proliferation (SI of 8.3±7.6), compared to mice immunized with LVSCE alone (SI of 3.8±0.06), and significantly higher production of IL-10 (*p*<0.05) and IL-17 (*p*<0.01) in response to ffLVS stimulation (Fig. 3). The stimulation index (14.9±6.6) for the LVSCE/CT immunized group was significantly higher (*p*<0.01 vs. LVSCE alone group). The IL-10 and lL-17 levels in the LVSCE/AMVAD and LVSCE/CT group were comparable (Fig. 3). Compared to the LVSCE group, the amount of IL-2 produced by the splenocytes from the LVSCE/AMVAD group was higher, but not significantly as seen with LVSCE/CT group. The IL-4 responses were below the detection limit (<10 pg/ml) in all groups of mice. It was somewhat surprising to note that compared to the LVSCE group, the IFN-γ levels were significantly different (*p*<0.05) and higher in the naïve group only. The IFN-γ levels seen with splenocytes from the LVSCE/CT and LVSCE/AMVAD groups were lower, being similar to the LVSCE group. In response to Con A stimulation (positive stimulation control), there were no significant differences in the magnitude of lymphocyte proliferation (SI of 8.2–10.5) or most of the cytokine levels (data not shown) between the different groups of mice.

**Figure 4:**
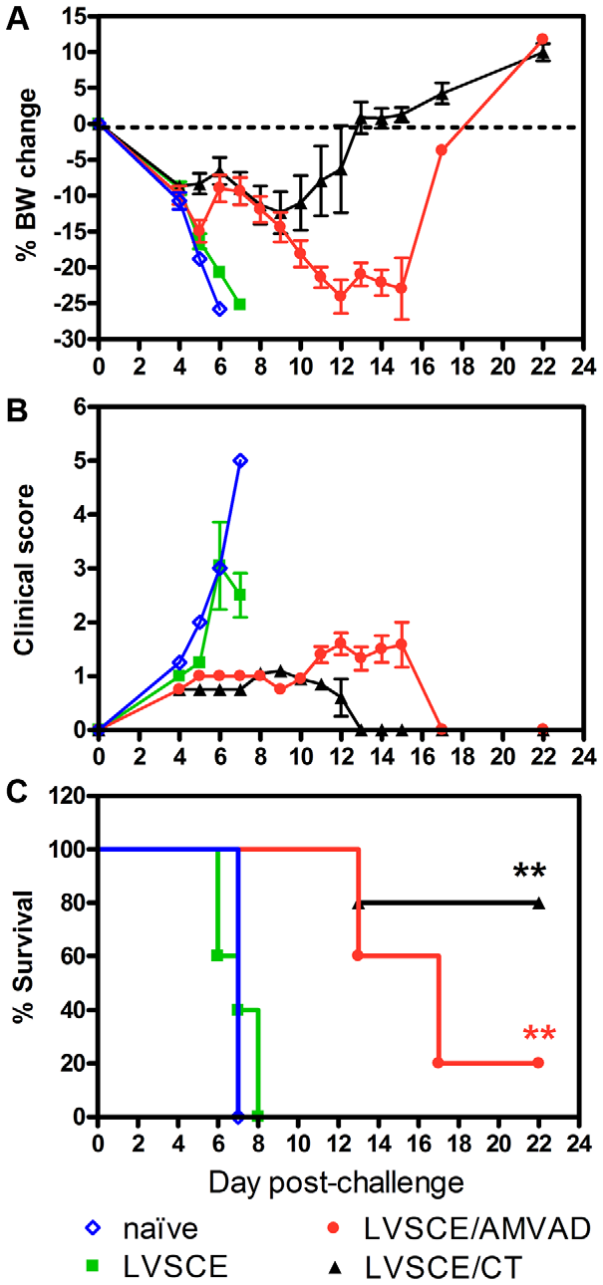
LVSCE/AMVAD vaccine partially protects against i.n. *F. tularensis* LVS challenge. The remaining naïve, and LVSCE, LVSCE/AMVAD or LVSCE/CT immunized groups of mice (n = 15) in Figure 1 experiment, were intranasally challenged with *F. tularensis* LVS (3.4×10^4^ cfu) at 35 d (14 d post the last immunization). The % change in body weight (A), clinical scores (B) and % survival (C) of sub-groups of 5 mice per each group was recorded over a 22 d period subsequent to the challenge. The data are presented as mean ± SD for each group (n = 5) for panels A and B. ^**^ *p*<0.01 vs. LVSCE alone immunized group. Data are representative of two separate experiments.

### Pre-challenge BAL cellular and cytokine profiles

At day 28 (7 d post third immunization), there were significantly higher numbers of total BAL cells and the numbers of alveolar macrophages in BAL fluids from the LVSCE/AMVAD (*p*<0.05) and LVSCE/CT (*p*<0.01) immunized groups, as compared to those in the LVSCE alone immunized or the naïve groups (Table 1). The numbers of neutrophils and lymphocytes in the BAL fluids of the LVSCE/AMVAD and LVSCE/CT immunized mice were also higher than in the antigen alone immunized (LVSCE) or naïve groups, but these were significantly higher (*p*<0.01) only in the LVSCE/CT immunized group. As expected, the BAL fluid cells from naïve mice were almost entirely comprised of macrophages (Table 1).

**Table 1:** Cell populations in the bronchoalveolar lavage fluids of mice at 28 d (7 d post third immunization at 21 d).

Group	Total number of cells ( ×10^5^)	Mean number of cells ( ×10^5^) and % of		
		Macrophages	Neutrophils	Lymphocytes
Naїve	7.50±3.31	7.38±3.28 (98±1%)	0.04±0.05 (0±1%)	0.09±0.02 (1±1%)
LVSCE	3.03±1.61	2.67±1.65 (86±9%)	0.09±0.04 (4±3%)	0.23±0.13 (9±6%)
LVSCE/AMVAD	15.38±6.93*	12.24±5.55* (80±6%)	0.75±0.67 (5±4%)	2.37±1.60 (14±6%)
LVSCE/CT	41.70±0.28**	26.87±9.21** (63±9%)	6.58±0.71** (16±4%)	8.25±2.75** (20±7%)

Data are presented as mean ± SD.

^*^ *p*<0.05 and ^**^ *p*<0.01 vs. LVSCE alone immunized group.

In the BAL fluids, we also measured the levels of a panel of 11 cytokines/chemokines that are known to be involved in the recruitment and activation of innate and acquired immune cells. Of these, KC (a neutrophil chemotactic factor) was significantly higher (*p*<0.01) in the BAL fluids of mice immunized with LVSCE/AMVAD (150±47 pg/ml) or LVSCE/CT (256±55 pg/ml), as compared to the naïve (34±8 pg/ml) or LVSCE alone immunized (66±22 pg/ml) groups. The IL-17 level was just above the detectable limit in the LVSCE/CT group only. The IP-10 levels in the BAL fluids of the LVSCE alone (74±33 pg/ml) and LVSCE/CT (88±24 pg/ml) groups were comparable, but these were significantly lower in the LVSCE/AMVAD (30±12 pg/ml) and naïve (16±8 pg/ml) groups, as compared with the LVSCE group. There was no detectable amount IFN-γ, IL-10, IL-12p70, IL-4, IL-6, MCP-1, RANTES or TNF-α (<10 pg/ml) in the BAL fluid of any mouse group (data not shown).

### Body weight, clinical scores and survival after i.n. *F. tularensis* LVS challenge

All groups of mice lost about 10% of their pre-challenge body weight by 4 days post the i.n. challenge with *F. tularensis* LVS (Fig. 4A). At 6–7 days post-challenge, the surviving mice in the LVSCE alone and naïve groups had lost close to 25% of their body weight and were euthanized (humane end point in conjunction with the clinical scores). In contrast, the LVSCE/CT immunized mice lost about 12% of their body weight by 9 days post-challenge, and the surviving mice began to gradually regain weight to achieve a 10% gain over the pre-challenge weight by 22 days post-challenge. The LVSCE/AMVAD immunized mice gradually lost weight for about 12 days post-challenge, and after a stabilization period of about 4 more days, the surviving mice began to regain the lost weight, achieving a gain similar to the LVSCE/CT group.

Based on the clinical scores (Fig. 4B), the naïve and LVSCE alone immunized mice began to get sick rapidly, and by 7–8 days post-challenge all of the mice were either dead or had to be euthanized due to high clinical scores and body weight loss. The mean clinical score of the LVSCE/AMVAD group at up to 10 days post-challenge was about 1.0 or lower, indicating mild sickness. Subsequently, the average clinical score increased to about 1.5 by 13 days post-challenge, and the scores for the surviving mice at that period began to stabilize and eventually reverted to 0 (fully normal) at 17 days post-challenge and beyond. The mice in the LVSCE/CT immunized group had a mean clinical score of less than 1.0 over the 13 days post-challenge, and then their health improved to achieve a clinical score of 0 at subsequent days.

All the mice in the LVSCE alone and naïve groups had died or had to be euthanized by day 8 post-challenge (Fig. 4C). In contrast, 100% of the mice in the LVSCE/AMVAD and LVSCE/CT immunized groups were alive at 12 days post-challenge. Significantly (*p*<0.01), 80% of the LVSCE/CT and 20% of the LVSCE/AMVAD immunized mice survived the ca 34× LD_50_ i.n. challenge with *F. tularensis* LVS, compared to the naïve or LVSCE immunized groups (Fig. 4C). Although a higher percentage of the LVSCE/CT immunized group survived the challenge compared to the LVSCE/AMVAD group, there were no statistically significant differences between the survival curves of these two groups.

### Lung and spleen pathogen burdens at 1 and 4 days post i.n. *F. tularensis* LVS challenge

**Figure 5:**
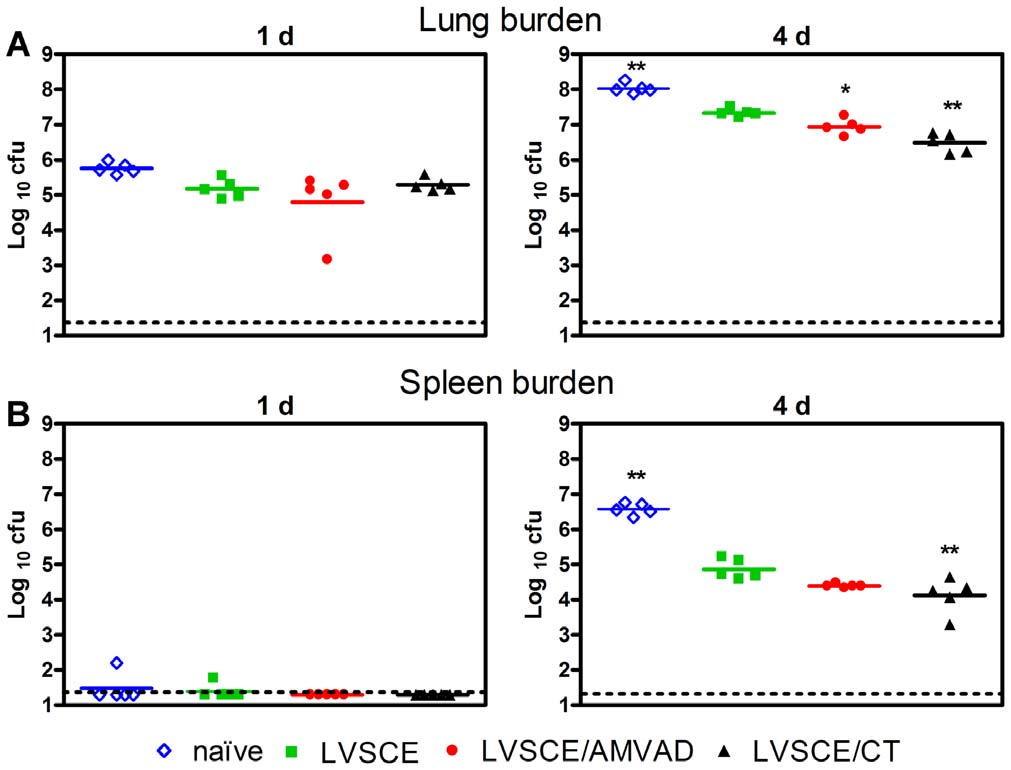
LVSCE/AMVAD vaccine reduces the bacterial burdens in the lungs and spleen. Five mice per naïve, and LVSCE, LVSCE/AMVAD or LVSCE/CT immunized groups challenged with 3.4×10^4^ cfu of *F. tularensis* LVS in the Figure 4 experiment were euthanized at 1 or 4 d post-challenge, and the *F. tularenisis* burden (log_10_ cfu/organ) in the lungs (A) and spleen (B) determined. The data are presented as mean log_10_ cfu ± SD for each group, at each time point (n = 5). The dotted line represents the detection limit. ^*^ *p*<0.05 and ^**^ *p*<0.01 vs. LVSCE alone immunized group. Data are representative of two separate experiments.

To determine if the AMVAD vaccine promoted pulmonary clearance of *F. tularensis* LVS and restricted the systemic dissemination of the bacterium, quantitative bacteriology was performed on the lungs and spleens at 1 and 4 day post-challenge. At 1 day post-challenge, the mean pathogen burdens in the lungs of all groups of mice were comparable (Fig. 5A), and the burdens in the spleens were generally below the detection limit (Fig. 5B). At 4 days post-challenge, the pathogen burdens in the lungs of LVSCE/AMVAD (*p*<0.05) and LVSCE/CT (*p*<0.01) groups were significantly lower (ca 0.5–1.0 log_10_) than those in the group immunized with LVSCE alone (Fig. 5A). At 4 days post-challenge, the pathogen burdens in the spleens of mice from the LVSCE/AMVAD group were lower (ca 0.5 log_10_), although not significantly, than in the LVSCE group (Fig 5B). The highest pathogen burdens in the lungs and spleen were seen in the naïve group.

### Serum and lung cytokine/chemokine profiles at 1 and 4 days post intranasal *F. tularensis* LVS challenge

**Figure 6:**
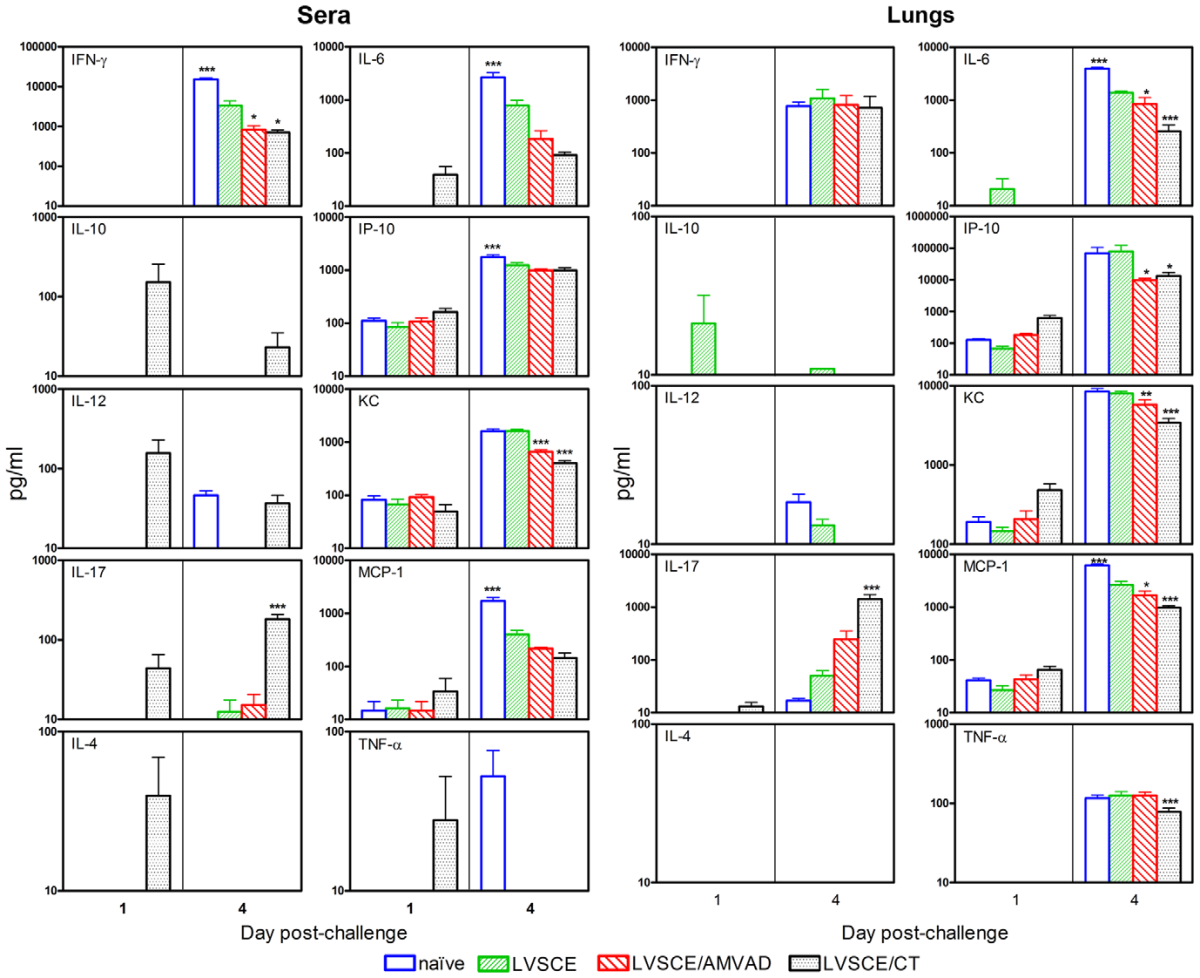
Changes in cytokine/chemokine levels in the sera (two left panels) and lungs (two right panels) subsequent to challenge. The sera and lung homogenates from naïve, and LVSCE, LVSCE/AMVAD or LVSCE/CT immunized groups of mice intranasally challenged with *F. tularensis* LVS (3.4×10^4^ cfu) and euthanized in Figure 5 experiment at 1 and 4 d post-challenge, were analyzed for the indicated cytokines/chemokines. The data are presented as mean ± SD for each group (n = 5). The assay detection limit is 10 pg/ml. ^*^ *p*<0.05, ^**^ *p*<0.01 and ^***^ *p*<0.001 vs. LVSCE alone immunized group.

A panel of 10 pro-inflammatory and T cell cytokines that were previously implicated in the pathogenesis of, and protection against, LVS infection were analyzed in the sera and lungs of mice at 1 and 4 days post-challenge (Fig. 6). At 1 day post-challenge, small to moderate, but comparable, amounts of IP-10, KC and MCP-1 were detected in the serum samples of all groups of mice. The serum levels of other cytokines/chemokines in all groups were generally below the detection limit (<10 pg/ml) at 1 day post-challenge, with the exception of IL-4, IL-6, IL-10, IL-12, IL17, and TNF-α in the LVSCE/CT group. The IFN-γ level was below the detection limit in all groups of mice at this time point. The serum levels of the majority of cytokines/chemokines (except IL-4, IL-10, and IL-12) increased dramatically at 4 days post-challenge, and the levels of IFN-γ, IL-6, IP-10 and MCP-1 were significantly higher (p<0.01) in the naïve group as compared to the LVSCE alone immunized group. At 4 days post-challenge, the levels of IFN-γ in the LVSCE/AMVAD and LVSCE/CT group were significantly lower (*p*<0.05) than that in the LVSCE group. IL-17 was the only cytokine whose level was significantly higher (*p*<0.001) in LVSCE/CT immunized group, as compared to the LVSCE alone group (Fig. 6).

Similar to the observations in sera, the majority of cytokines/chemokines were below the detection limit in the lung homogenates at 1 day post-challenge, with the exception of moderate and comparable amounts of IP-10, KC and MCP-1 detected in all groups of mice (Fig. 6). The levels of IFN-γ, IL-17, IL-6, IP-10, KC, MCP-1 and TNF-α in the lungs of all mice were substantially higher at 4 days post-challenge, as compared to at 1 day (Fig. 6). The levels of IL-10 and IL-4 were at or below the detection limit (<10 pg/ml). As in the serum, most of the BAL cytokine/chemokine levels at this time point were higher in the naïve mice and mice immunized with LVSCE alone, than those in mice immunized with LVSCE/CT or LVSCE/AMVAD, with the exception of the IL-17. The level of IL-17 was higher in the LVSCE/AMVAD group and significantly higher (*p*<0.001) in the LVSCE/CT immunized group, as compared to the LVSCE alone immunized group. There were no significant differences in the IFN-γ levels in of all groups of mice, compared to the LVSCE alone group (Fig. 6).

## Discussion

In this study, we evaluated the ability of the AMVAD system to elicit protective immunity against mucosal infections, using a mouse model of i.n. *F. tularensis* LVS challenge. Compared with LVSCE antigen alone, the LVSCE/AMVAD vaccine induced substantially higher mucosal and systemic antibody responses. The LVSCE/AMVAD vaccine also induced cellular immune responses, as assessed by antigen-specific lymphocyte proliferation and the production of several T cell cytokines such as IL-17 and IL-10. More importantly, the immune responses induced by the LVSCE/AMVAD vaccine partially protected against a 34× LD_50_ i.n. challenge with *F. tularensis* LVS, as judged from the substantially to significantly reduced pathogen burdens in the lungs and spleens, an increased mean time to death and a higher survival rate, as compared to mice immunized with LVSCE antigen alone.

Cholera toxin was used as a positive control adjuvant in this work since it represents one of the most potent and frequently used experimental mucosal adjuvants [8]. The antibody responses induced by LVSCE/AMVAD immunization were generally robust, but the responses induced by LVSCE/CT were usually stronger. However, the considerable toxicity of CT in humans precludes its direct application in mucosal vaccines for humans.

Although our results demonstrated that LVSCE/AMVAD is capable of inducing protective immune responses against a lethal i.n. challenge with *F. tularensis* LVS, the precise mechanism responsible for this immunity remains to be characterized. The anti-LVSCE IgA antibody responses in the sera and BAL fluid of the LVSCE/AMVAD and LVSCE/CT groups were much stronger (and somewhat comparable) compared to the little response in the LVSCE or none in the naïve group. Since the LVSCE/AMVAD and LVSCE/CT were the only groups showing increased time to death and partial survival upon an i.n. *F. tularensis* LVS challenge, it suggests that mucosal IgA plays some protective role in this challenge model. In this regard, it has been recently shown by several groups that mucosal IgA is important in host defense against infections with *F. tularensis* and *F. novicida* [20], [21], [22].

Although it is generally recognized that cell-mediated immunity (CMI) is required for protection against an infection with the more virulent clinical type A and type B strains of *F. tularensis* [23], it is less clear whether or not CMI is imperative for host defense against infection with the less virulent *F. tularensis* LVS. However, studies by several groups have previously shown that specific antibodies or serum transfer appear to be sufficient for protection against systemic LVS challenge [24], [25], [26]. The antigen-specific IgG2a antibody titer was higher in the sera from the LVSCE/CT immunized group, compared to that in the LVSCE/AMVAD and LVSCE groups. Since LVSCE/CT-immunized group also had a higher, but not significantly, survival rate against the i.n. LVS challenge than did the LVSCE/AMVAD group, it is plausible to speculate that in addition to the mucosal IgA, IgG2a plays a key role in the host defense against i.n. LVS challenge. In this regard, it is well established that IgG2a can function as an important effector molecule in the antibacterial activities through its role in the antibody-dependent cell-mediated cytotoxicity and complement fixation [27]. In addition, a robust IgG2a response could be regarded as an indication of a strong induction of a Th1-biased immune response, which in turn could play a crucial role in host defense against *F. tularensis* infection. In this regard, we noted that LVSCE/AMVAD or LVSCE/CT immunization appears to induce no significant increase in the production of Th1 cytokines IFN-γ and IP-10 by splenocytes compared to the LVSCE group, but the LVSCE/CT group did produce significantly higher IL-2 level upon *in vitro* stimulation with ffLVS (Fig. 3). It should be noted that the weaker IgG2a response seen with LVSCE/AMVAD vaccine in this study does not suggest an inherent deficiency of the AMVAD system, and appears to be related to the specific LVSCE antigen, since we have seen that intranasally administered OVA/AMVAD [13] and other antigen/AMVAD (unpublished data) vaccines induce strong antigen-specific IgG2a responses compared to the antigen alone groups.

To further understand the protective mechanism induced by LVSCE/AMVAD vaccine, we compared the *in vitro* cytokine/chemokine production in response to antigen stimulation of splenocytes from mice immunized with LVSCE/AMVAD and LVSCE alone. We found that the splenocytes from LVSCE/AMVAD immunized mice produced significantly higher amounts of IL-17 and IL-10 and lower amount of IFN-γ in response to ffLVS stimulation compared to the mice immunized with LVSCE alone or naïve mice (Fig. 3). In this regard, it is interesting to note that several groups have recently shown that endogenous IL-17 plays a crucial role in host defence against *F. tularensis* LVS infection and IL-17−/− mice are incapable of controlling i.n. LVS challenge [28], [29], [30]. Thus, the enhanced IL-17 production seen in LVSCE/AMVAD and LVSCE/CT immunized mice may partially account for their enhanced protection against the i.n. *F. tularensis* LVS challenge. On the other hand, it was somewhat surprising to note that both the LVSCE/AMVAD and LVSCE/CT immunization appeared to have suppressed antigen-specific IFN-γ responses. However, it is possible that the high IFN-γ response seen in naïve mice (Fig. 3) was probably related to the non-specific response to whole cell antigen preparation (ffLVS) that we used in the assay. Although IFN-γ is a key cytokine in host defence against almost all intracellular pathogens including *F. tularensis*, this cytokine has been shown to be more critical for the control of primary rather than secondary infection with *F. tularensis* LVS [31]. In addition, compared to systemic infection, IFN-γ appears to play a less crucial role in the control of *F. tularensis* LVS infection via the respiratory route [32]. Thus, it is possible that IFN-γ played a marginal protective role in the current model.

We also monitored the cytokine/chemokine levels in sera and lungs of the immunized and naïve mice after i.n. challenge with lethal doses of *F. tularensis* LVS (Fig. 6). With the exception of IL-17 which was significantly higher in the LVSCE/CT group as compared to the naïve or LVSCE alone groups, the cytokine/chemokine levels in both the lungs and sera were generally either similar or lower in the more protected LVSCE/CT and LVSCE/AMVAD groups, as compared to the naïve or LVSCE alone group. Thus, IL-17 was the only cytokine that distinguished between the highly protected LVSCE/CT group versus the unprotected naïve and the LVSCE alone immunized groups. There was little correlation between serum and lung levels of other cytokines and the protective efficacy induced by the LVSCE/AMVAD vaccine, and their levels seem to reflect the tissue bacterial burdens (antigen loading) and the extent of infection.

In summary, i.n. immunization of vaccine adjuvanted with the AMVAD system induces antigen-specific mucosal and systemic antibody and CMI responses, and protects mice against a lethal i.n. *F. tularensis* LVS challenge. The possible roles of IgA and IgG2a antibody responses, and of IL-17, in the protective efficacy are implied. The AMVAD system elicits long-lasting and memory boostable mucosal and systemic immune responses [13] and preclinical murine studies have shown it to be safe [14]. It is possible that with further experimentation regarding the antigen dose, antigen/adjuvant ratio, the immunization schedule, or the use of a specific identified protective antigen, the efficacy of the AMVAD adjuvanted vaccine in the i.n. *F. tularensis* LVS challenge model could be enhanced further. The current findings warrant additional exploration of the AMVAD system as an alternative mucosal adjuvant/vaccine delivery technology, for developing vaccines against mucosal pathogens.
